# OAO1.01. Are complementary therapies and integrative care cost-effective? A comprehensive systematic review of economic evaluations

**DOI:** 10.1186/1472-6882-12-S1-O1

**Published:** 2012-06-12

**Authors:** P Herman, B Poindexter, C Witt, D Eisenberg

**Affiliations:** 1 University of Arizona, Tucson, USA; 2 Charité University Medical Center, Berlin, Germany; 3 Harvard Medical School and Harvard School of Public Health, Boston, USA

## Purpose

One-third or more US adults, and similar numbers elsewhere, use complementary and integrative medicine (CIM). The results of previous systematic reviews of the economic impacts of CIM are limited and now out of date. The purpose of this comprehensive review is to capture and highlight for policy makers what is currently known about the economics of CIM, and to make recommendations for future research.

## Methods

PubMed, CINAHL, AMED, PsychInfo, Web of Science, and EMBASE were searched through December 2010 using a comprehensive search strategy. In addition, bibliographies were searched, and key researchers contacted. All full economic evaluations published 2001-2010 were also subjected to five study quality criteria and one indicator of whether the study’s results were transferable (i.e., could be adjusted to apply/generalize to other settings).

## Results

A total of 340 economic evaluations of CIM were found, of which 206, covering a wide variety of CIM for different populations, were published 2001-2010, and 134 of those were full economic evaluations. Despite the fact that most CIM users utilize more than one type of CIM, almost all (88%) of these studies were of single CIM therapies and only one evaluated coordinated care across CIM and conventional practitioners. Of the recent full evaluations 32 (24%) met all five study quality criteria, and 17 of these also met the minimum criterion for study transferability.

## Conclusion

This comprehensive review identified a substantial number of economic evaluations of CIM and emerging evidence of cost-effectiveness in at least a few clinical populations. Therefore, instead of more studies, what is actually needed are higher quality studies—both in terms of enhanced study quality (to increase the validity of the results for its targeted population and setting) and better transferability (to increase the usefulness of results to other decision-makers in other settings). To this end, eight specific recommendations are made.

